# Predictive Value of HFA-PEFF Score in Patients With Heart Failure With Preserved Ejection Fraction

**DOI:** 10.3389/fcvm.2021.656536

**Published:** 2021-10-29

**Authors:** Yuxi Sun, Jinping Si, Jiaxin Li, Mengyuan Dai, Emma King, Xinxin Zhang, Yanli Zhang, Yunlong Xia, Gary Tse, Ying Liu

**Affiliations:** ^1^Heart Failure and Structural Cardiology Ward, First Affiliated Hospital of Dalian Medical University, Dalian, China; ^2^Cardiovascular Analytics Group, Hong Kong SAR, China; ^3^Kent and Medway Medical School, Canterbury, United Kingdom

**Keywords:** predictive value, all-cause mortality, HFpEF, HFA-PEFF score, prognosis

## Abstract

**Aims:** HFA-PEFF score has been proposed for diagnosing heart failure with preserved ejection fraction (HFpEF). Currently, there are only a limited number of tools for predicting the prognosis. In this study, we evaluated whether the HFA-PEFF score can predict mortality in patients with HFpEF.

**Methods:** This single-center, retrospective observational study enrolled patients diagnosed with HFpEF at the First Affiliated Hospital of Dalian Medical University between January 1, 2015, and April 30, 2018. The subjects were divided according to their HFA-PEFF score into low (0–2 points), intermediate (3–4 points), and high (5–6 points) score groups. The primary outcome was all-cause mortality.

**Results:** A total of 358 patients (mean age: 70.21 ± 8.64 years, 58.1% female) were included. Of these, 63 (17.6%), 156 (43.6%), and 139 (38.8%) were classified into the low, intermediate, and high score groups, respectively. Over a mean follow-up of 26.9 months, 46 patients (12.8%) died. The percentage of patients who died in the low, intermediate, and high score groups were 1 (1.6%), 18 (11.5%), and 27 (19.4%), respectively. A multivariate Cox regression identified HFA-PEFF score as an independent predictor of all-cause mortality [hazard ratio (*HR*):1.314, 95% *CI*: 1.013–1.705, *P* = 0.039]. A Cox analysis demonstrated a significantly higher rate of mortality in the intermediate (*HR*: 4.912, 95% *CI* 1.154–20.907, *P* = 0.031) and high score groups (*HR*: 5.291, 95% *CI*: 1.239–22.593, *P* = 0.024) than the low score group. A receiver operating characteristic (ROC) analysis indicated that the HFA-PEFF score can effectively predict all-cause mortality after adjusting for age and New York Heart Association (NYHA) class [area under the curve (AUC) 0.726, 95% *CI* 0.651–0.800, *P* = 0.000]. With an HFA-PEFF score cut-off value of 3.5, the sensitivity and specificity were 78.3 and 54.8%, respectively. The AUC on ROC analysis for the biomarker component of the score was similar to that of the total score.

**Conclusions:** The HFA-PEFF score can be used both to diagnose HFpEF and predict the prognosis. The higher scores are associated with higher all-cause mortality.

## Introduction

Heart failure (HF) represents the final common pathway of different cardiac diseases. It is a rising global epidemic with an estimated prevalence of >37.7 million individuals globally (1). The patients with HF experience various symptoms, including dyspnea, poor exercise tolerance, and fluid retention, and have a poor long-term prognosis. The latest European Society of Cardiology (ESC) guidelines divided HF involving left ventricular ejection fraction (LVEF) into HF with reduced ejection fraction (HFrEF), HF with mid-range ejection fraction (HFmrEF), and HF with preserved ejection fraction (HFpEF) (2). Previous work reported that in the general population aged ≥ 60 years, 4.9% were identified as having HFpEF in Europe (3). This number is expected to increase with an aging population (4). Indeed, the HFpEF is usually considered to evolve from a combination of risk factors and comorbidities, including advanced age, female gender, obesity, hypertension, diabetes mellitus, renal dysfunction, anemia, iron deficiency, sleep disorders, and chronic obstructive pulmonary disease (5–8). Major mechanisms affecting the myocardium in HFpEF include left atrial hypertension, pulmonary hypertension, plasma volume expansion, systemic microvascular inflammation, cardiometabolic functional abnormalities, and cellular/extracellular structural abnormalities (9). It must be pointed out that before the publication of the PARALLAX trial, there was no convincing evidence-based strategy that has been shown to improve prognosis in patients with HFpEF.

Pieske et al. proposed the HFA-PEFF diagnostic algorithm for HFpEF in 2019 (10). Two points were allocated for major criteria and one point was allocated for minor criteria in the functional, morphological, and biomarker domains. The calculations and interpretations are as follows: ≤ 1 point (HFpEF unlikely); 2–4 points (diagnostic uncertainty and need further evaluation) and ≥5 points (definite HFpEF). While this score has been well-validated for the diagnosis of HFpEF, its relevance to the prognosis remains unclear. Therefore, we further investigated the predictive value of the score in this cohort study of the patients with HFpEF.

## Materials and Methods

### A Detailed Description of the HFA-PEFF Score

The HFA-PEFF diagnostic algorithm contains four consecutive steps. Step 1: initial workup, including assessment of the symptoms and signs of HF, the comorbidities or risk factors, ECG, echocardiography, natriuretic peptides, 6 min walking test, or cardiopulmonary exercise testing. Step 2: diagnostic workup, including echocardiography, and natriuretic peptide score. Step 3: advanced workups (functional testing in the case of uncertainty), such as exercise stress echocardiography and invasive hemodynamic measurements. Step 4: etiological workup, comprising cardiovascular magnetic resonance, cardiac or non-cardiac biopsies, scintigraphy or CT or PET, genetic testing, and specific laboratory tests. The specific echocardiographic variables include mitral annular early diastolic velocity (e'), left ventricular (LV) filling pressure (E/e'), left atrial volume index, LV mass index, LV relative wall thickness, tricuspid regurgitation velocity, LV global longitudinal systolic strain, and natriuretic peptide levels. Additionally, they recommended a scoring system based on the functional, morphological and biomarker domains (Table 1). If any major criterion from this domain is positive, this domain can contribute 2 points; if no major but any minor criterion is positive, this domain contributes 1 point. If several major criteria within a domain are positive, this domain still contributes 2 points; and if no major, but several minor criteria are positive the contribution is still 1 point. Notably, the HFA-PEFF score can be calculated even if all the parameters are not obtained, which can add to the utility of the score in clinical practice.

**Table 1:** Echocardiography and natriuretic peptides scoring system.

**Score**	**Functional**	**Morphological**	**Biomarker (sinus rhythm)**	**Biomarker (atrial fibrillation)**
Major criteria	1. septal e' <7 cm/s or lateral e' <10 cm/s or 2. Average E/e' ≥ 15 or 3. TR velocity > 2.8 m/s (PASP > 35 mmHg)	1. LAVI > 34 ml/m^2^ or 2. LVMI ≥ 149/122 g/m^2^(m/w) and RWT > 0.42	NT-proBNP > 220 pg/ml or BNP > 80 pg/ml	NT-proBNP > 660 pg/ml or BNP > 240 pg/ml
Minor criteria	1. Average E/e' 9–14 or 2. GLS <16%	1. LAVI 29–34 ml/m^2^ or 2. LVMI > 115/95 g/m^2^ (m/w) or 3. RWT > 0.42 or 4. LV wall thickness ≥ 12 mm	NT-proBNP 125–220 pg/ml or BNP 35–80 pg/ml	NT-proBNP 365–660 pg/ml or BNP 105–240 pg/ml

*e', mitral annular early diastolic velocity; E/e', mitral Doppler early velocity/ mitral annular early velocity; TR velocity, tricuspid regurgitation peak velocity; PASP, pulmonary arterial systolic pressure; GLS, global longitudinal strain; LAVI, left atrial volume index; LVMI, left ventricular mass index; RWT, relative wall thickness; NT-proBNP, N-terminal pro b-type natriuretic peptide; BNP, B-type natriuretic peptide*.

### Study Population and Groups

This study was a retrospective, single-center, observational study, and was approved by the institutional review board of Dalian Medical University, Liaoning, China. All the procedures were conducted in accordance with the Declaration of Helsinki and its amendments. In this study, 856 consecutive patients with HFpEF were hospitalized at the First Affiliated Hospital of Dalian Medical University between January 1, 2015, and April 30, 2018. The exclusion criteria were missing echocardiographic results, loss to follow-up, severe valvular heart disease, or end-stage renal failure. The cohort was divided into three groups based on the score: low (0–2 points), intermediate (3–4 points), and high (5–6 points) score groups.

### Clinical Definitions

Heart failure with preserved ejection fraction was defined according to the ESC guidelines: (i) symptoms or signs; (ii) LVEF ≥ 50%; (iii) elevated levels of natriuretic peptides, and at least one of the additional criteria: (1) relevant structural heart disease (LV or left atrial enlargement); (2) diastolic dysfunction (2). Hypertension was defined as a recorded blood pressure ≥ 140/90 mmHg or any prescription of antihypertensive medications (11). Diabetes mellitus was defined as treatment with any antidiabetic medication, fasting plasma glucose ≥ 7 mmol/L, or HbA1C ≥ 6.5%, or the presence of symptoms of diabetes and a random plasma glucose concentration ≥ 11.1 mmol/L (12).

### Clinical Data

Details of the clinical characteristics, drug therapy, comorbidities, biomarker assessment, arrhythmias, and echocardiography findings were collected and recorded from Yidu Cloud. The laboratory indicators were measured on admission, with a requirement to fast for more than 8 h before venous blood collection. All the subjects underwent dynamic electrocardiography to record various arrhythmias. Echocardiography was performed with the patients at rest, before discharge by the experienced cardiologists.

### Endpoint and Follow-Up

The primary outcome was all-cause mortality. After discharge, all the patients were required to return to the outpatient department regularly. If the patients did not attend their scheduled clinic appointments, they were interviewed by telephone annually. The cutoff was November 30, 2018, or the occurrence of death.

### Statistical Analysis

Statistical analysis was performed with Statistical Package for Social Sciences, version 24.0 (SPSS Inc., Chicago, IL, USA). Counting data were expressed as percentages (%), and the chi-squared test was used for comparison among the three groups. Measurement data with non-normal distribution were expressed as the median (interquartile range), and the Kruskal–Wallis test was used to assess the differences among the groups. The quantitative variables of normal distribution were expressed as arithmetic means ± SDs (x ± s), and ANOVA was used for between-group comparisons. A Kaplan–Meier analysis was performed to calculate the cumulative incidence of all-cause mortality, and the log-rank test was used to compare the differences. A Cox regression analysis was used to investigate the risk factors of adverse events in the patients with HFpEF. The HRs with 95% *CI*s were presented. A ROC curve was constructed from logistic regression to assess the availability of the HFA-PEFF score to predict the all-cause mortality in the patients with HFpEF. All the values were two-tailed, and a *p*-value <0.05 was considered statistically significant.

## Results

**Figure 1:**
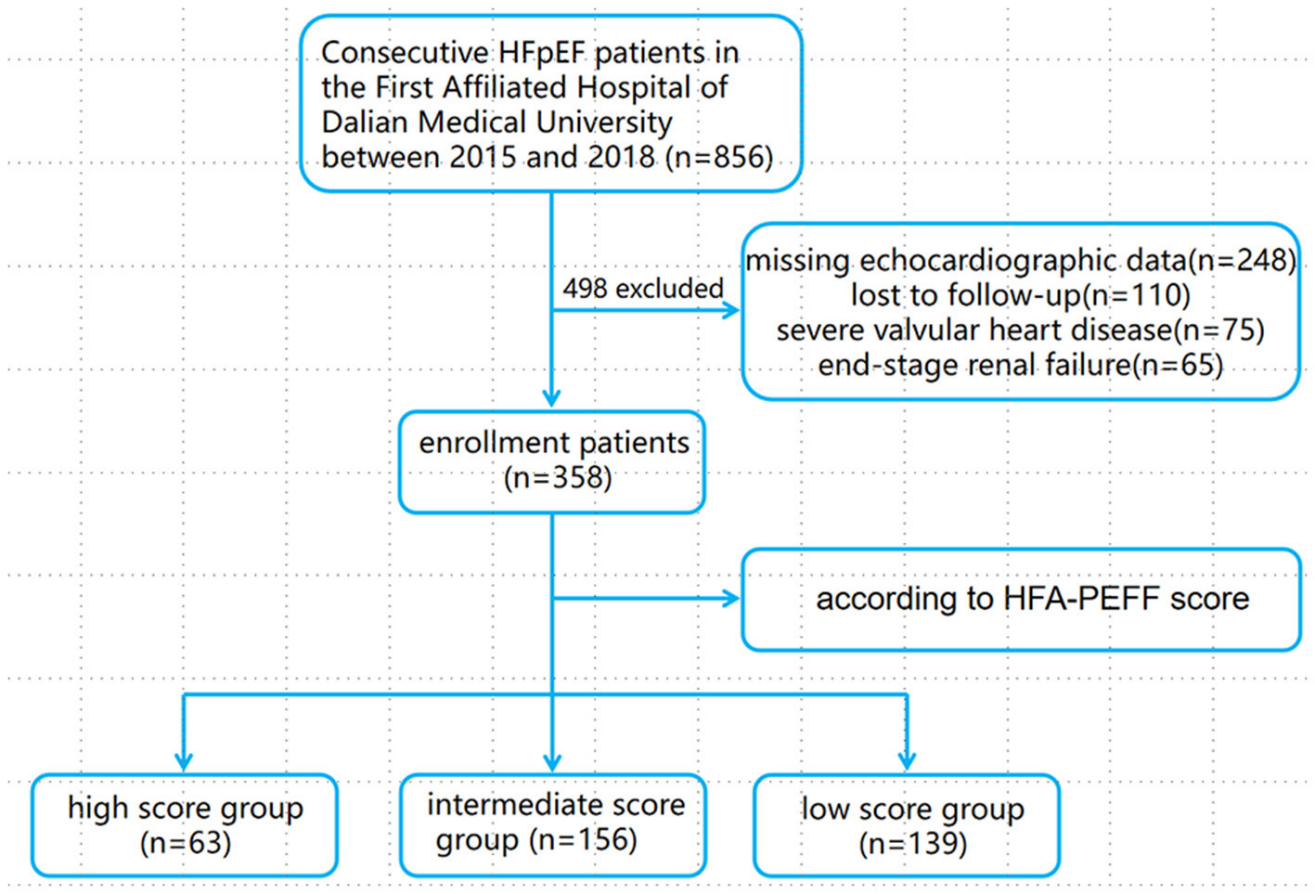
Flowchart of the study protocol.

A total of 856 consecutive patients with HFpEF who were hospitalized at the First Affiliated Hospital of Dalian Medical University between January 1, 2015, and April 30, 2018, were initially identified. Of these, 498 patients were excluded due to missing echocardiographic results (*n* = 248), loss to follow-up (*n* = 110), severe valvular heart disease (*n* = 75), or end-stage renal failure (*n* = 65). In the remaining cohort, the percentages of the low, intermediate, and high score groups were 63 (17.6%), 156 (43.6%), and 139 (38.8%), respectively. The study flowchart is shown in Figure 1.

### Baseline Characteristics

The mean age of the subjects was 70.21 ± 8.64 years, with 58.1% female. The baseline characteristics are shown in Table 2. Overall, the patients in the high score group had a faster heart rate (*P* < 0.001), were more likely to have a history of atrial fibrillation (AF)/flutter (*P* < 0.001), more often took medications, such as loop diuretics (*P* = 0.039) and digoxin (*P* = 0.025). In contrast, the patients in the low score group had a heavier burden of myocardial infarction (MI) (*P* = 0.020), higher surgical rates of percutaneous coronary intervention (PCI) (*P* = 0.043), and more often took medicines, such as statin (*P* = 0.011) and antiplatelet drugs (*P* < 0.001). In terms of laboratory data, the patients in the high score group had a higher level of B-type natriuretic peptide (BNP) (*P* < 0.001), high sensitivity troponin (hs-TNI) (*P* < 0.001), and serum potassium (*P* = 0.025) but lower level of hemoglobin (*P* = 0.010), triglyceride (*P* = 0.013), cholesterol (*P* = 0.002), high density lipoprotein cholesterol (HDL-C) (*P* < 0.001), and low density lipoprotein cholesterol (LDL-C) (*P* = 0.023) compared with those in the low and intermediate score groups. Interestingly, the discrepancy in the natriuretic peptides was mainly observed in the patients with sinus rhythm (SR) rather than those with AF. Regarding echocardiographic findings, the high score group had higher values of left atrial volume index (LAVI) (*P* < 0.001), LV wall thickness (*P* = 0.01), LV end diastolic diameter (*P* < 0.001), mitral doppler early velocity/mitral annular early velocity (E/e') (*P* < 0.001), and lower early to late diastolic transmitral flow velocity (E/A) (*P* < 0.001) than the low and intermediate groups. Furthermore, the patients with AF had higher LAVI compared with those with SR. By contrast, no statistical differences among the three groups were observed for left ventricular mass index (LVMI) and relative wall thickness (RWT). In the subgroup analysis of LVMI, the intermediate group had the highest value than the low and high score groups in the male (*P* = 0.005), mainly due to the higher frequency of hypertrophic cardiomyopathy.

**Table 2:** The baseline characteristics.

	**Total**	**0–2 points**	**3–4 points**	**5–6 points**	***P*-value**
Case (*n*, %)	358	63 (17.6)	156 (43.6)	139 (38.8)	—
Age (years)	70.21 ± 8.64	70.05 ± 8.88	69.26 ± 9.54	71.34 ± 7.29	0.118
Female (*n*,%)	208 (58.1)	39 (61.9)	91 (58.3)	78 (56.1)	0.740
NYHA class > 2 (*n*, %)	287 (80.2)	47 (74.6)	128 (82.1)	112 (80.6)	0.452
Heart rate (bpm)	81.11 ± 21.09	73.71 ± 14.28^ψ^^†^	79.93 ± 20.82^*^^†^	85.78 ± 22.88^*^^ψ^	<0.001
BMI (kg/m^2^)	26.87 ± 3.96	27.38 ± 4.12	26.97 ± 3.74	26.63 ± 4.11	0.341
Systolic blood pressure (mmHg)	143.73 ± 24.97	149.14 ± 25.96	144.46 ± 26.92	140.47 ± 21.70	0.065
Diastolic blood pressure (mmHg)	81.68 ± 15.87	78.44 ± 15.72	82.51 ± 16.82	82.21 ± 14.73	0.203
QRS (ms)	81.68 ± 15.87	78.44 ± 15.72	82.51 ± 16.82	82.21 ± 14.73	0.079
QT (ms)	407.08 ± 53.89	421.18 ± 41.97^ψ^^†^	411.83 ± 56.03^*^	396.07 ± 54.20^*^	0.009
Atrial fibrillation /flutter (*n*, %)	191 (53.7)	0 (0.0)^ψ^^†^	52 (14.5)^*^^†^	139 (100.0)^*^^ψ^	<0.001
Hypertension (*n*, %)	289(80.7)	51 (81.0)	129 (82.7)	109 (78.4)	0.867
Diabetes (*n*, %)	175(48.9)	31 (49.2)	80 (51.2)	64 (46.0)	0.677
Prior MI (*n*, %)	84 (23.5)	23 (36.5)^ψ^^†^	35 (22.4)^*^	26 (18.7)^*^	0.020
Angina (*n*, %)	53(14.8)	13 (20.6)	25 (16.0)	15 (10.8)	0.161
Prior PCI (*n*, %)	39 (10.9)	12 (19.0)	17 (10.9)	10 (7.2)	0.043
Prior pacemakers (*n*, %)	14 (3.9)	4 (6.3)	6 (3.8)	4 (2.9)	0.531
**Laboratory test**					
Hemoglobin (g/L)	125.83 ± 25.96	135.61 ± 17.23^ψ^^†^	126.00 ± 24.43^*^	121.14 ± 29.61^*^	0.010
Hs-TnI (ug/L)	0.019 (0.006, 0.044)	0.009 (0.006, 0.022)^ψ^^†^	0.018 (0.006, 0.050)^*^^†^	0.028 (0.014, 0.047)^*^^ψ^	<0.001
D-Dimer (ug/ml)	570 (270, 1,210)	400 (230, 755)	525 (250, 1,215)	660 (388, 1,598)	0.393
Glu (mmol/L)	6.68 ± 2.78	6.85 ± 2.93	6.84 ± 3.06	6.41 ± 2.34	0.378
Cre (mmol/L)	79 (64, 103)	73 (60, 94)^ψ^^†^	81 (66, 106)^*^	82 (64, 109)^*^	0.010
UA (umol/L)	421 (514, 337)	395 (315, 512)	426 (324, 514)	479 (393, 639)	0.166
TC (mmol/L)	4.50 ± 1.20	4.93 ± 1.46^ψ^^†^	4.51 ± 1.20^*^	4.27 ± 1.00^*^	0.002
TG (mmol/L)	1.42 ± 0.80	1.67 ± 1.11^ψ^^†^	1.42 ± 0.74^*^	1.31 ± 0.66^*^	0.013
HDL-C (mmol/L)	1.13 ± 0.33	1.28 ± 0.45^ψ^^†^	1.14 ± 0.30^*^^†^	1.06 ± 0.27^*^^ψ^	<0.001
LDL-C (mmol/L)	2.53 ± 0.85	2.78 ± 1.07^†^	2.54 ± 0.85	2.41 ± 0.70^*^	0.023
Na (mmol/L)	141.72 ± 3.80	141.92 ± 3.67	141.96 ± 3.37	141.35 ± 4.30	0.356
K (mmol/L)	3.96 ± 0.50	3.81 ± 0.46^ψ^^†^	3.98 ± 0.48^*^	4.01 ± 0.54^*^	0.025
BNP (pg/ml)	275.00 (138.36, 524.48)	86.80 (47.91, 157.00)^ψ^^†^	257.78 (152.04, 484.44)^*^^†^	465.65 (283.38, 801.90)^*^^ψ^	<0.001
BNP in AF (pg/ml)	298.74 (178.09, 491.00)	142.00 (95.23, 174.18)	251.89 (164.42, 437.97)	431.70 (276.58, 614.06)	0.115
BNP in SR (pg/ml)	231.95 (81.96, 632.84)	58.22 (36.01, 109.47)^†^	274.25 (131.00, 573.44)^†^	587.83 (297.48, 1,136.00)^*^^ψ^	<0.001
**Echocardiographic parameters**					
Left atrial volume index (LAVI) (ml/m^2^)	35.73 ± 15.14	21.47 ± 4.62^ψ^^†^	33.12 ± 12.73^*^^†^	42.63 ± 12.00^*^^ψ^	<0.001
LAVI in AF (ml/m^2^)	40.72 ± 11.78	—	35.56 ± 9.54^†^	42.72 ± 11.99^ψ^	<0.001
LAVI in SR (ml/m^2^)	28.53 ± 10.24	22.89 ± 5.78^ψ^	31.86 ± 10.89^*^	—	<0.001
Left ventricular mass index (LVMI) (g/m^2^)	111.09 ± 30.27	106.92 ± 31.43	114.78 ± 34.23	108.90 ± 24.19	0.126
LVMI in male (g/m^2^)	114.91 ± 31.96	106.88 ± 36.72^ψ^	124.94 ± 33.20^*^^†^	107.91 ± 25.82^ψ^	0.005
LVMI in female (g/m^2^)	108.38 ± 28.77	106.94 ± 28.12	107.89 ± 33.36	109.68 ± 22.97	0.872
RWT (%)	0.48 ± 0.091	0.49 ± 0.078	0.48 ± 0.10	0.48 ± 0.083	0.593
Left ventricular wall thickness (mm)	10.58 ± 1.46	10.08 ± 1.55^ψ^^†^	10.67 ± 1.43^*^	10.72 ± 1.41^*^	0.010
E/e'	12.15 ± 5.45	8.27 ± 2.59^ψ^^†^	10.72 ± 4.10^*^^†^	15.52 ± 5.86^*^^ψ^	<0.001
E/A (%)	0.86 (0.63,1.47)	1.54 (1.17,1.71)^ψ^^†^	0.82 (0.64,1.51)^*^^†^	0.66 (0.54,0.85)^ψ^^†^	<0.001
Interventricular septal thickness (mm)	11.14 ± 2.42	11.47 ± 1.86	11.11 ± 2.74	11.03 ± 2.25	0.484
Left ventricular end diastolic diameter (mm)	47.57 ± 6.22	44.75 ± 5.63^ψ^^†^	47.50 ± 6.64^*^^†^	48.99 ± 5.54^*^^ψ^	<0.001
LVEF (*n*, %)	56.68 ± 3.46	58.02 ± 1.64^ψ^^†^	56.47 ± 2.67^*^	56.29 ± 4.54^*^	0.003
**Pharmacotherapeutics**					
Loop diuretics (*n*, %)	228 (63.7)	34 (54.0)^†^	95 (60.9)	99 (71.2)^*^	0.039
Beta-blockers (*n*, %)	263 (73.5)	43 (68.3)	119 (76.3)	101 (72.7)	0.459
ACEI (*n*, %)	109 (30.6)	19 (30.2)	48 (30.8)	42 (30.2)	0.993
ARB (*n*, %)	115 (32.3)	23 (36.5)	49 (31.4)	43 (30.9)	0.711
Spironolactone (*n*, %)	182 (51.1)	33 (52.4)	70 (44.9)	79 (56.8)	0.118
CCB (*n*, %)	138 (38.8)	24 (38.1)	62 (39.7)	52 (37.4)	0.897
Digoxin (*n*, %)	31 (8.7)	1 (1.6)^†^	12 (7.7)	18 (12.9)^*^	0.025
Statin (*n*, %)	221 (61.7)	47 (74.6)^†^	100 (64.1)	74 (53.2)^*^	0.011
Antiplatelet drug (*n*, %)	159 (44.4)	42 (66.7)^ψ^^†^	68 (43.6)^*^	49 (35.3)^*^	<0.001

*NYHA, New York Heart Association; BMI, body mass index; Prior MI, prior myocardial infarction; Prior PCI, prior percutaneous coronary intervention; hs-TNI, high sensitivity troponin; Glu, glucose; Cre, creatinine; UA, uric acid; TC, cholesterol; TG, triglyceride; HDL-C, high density lipoprotein cholesterol; LDL-C, low density lipoprotein cholesterol; Na, serum sodium; K, serum potassium; BNP, B-type natriuretic peptide; AF, atrial fibrillation; SR, sinus rhythm; RWT, relative wall thickness; E/e′, mitral Doppler early velocity/mitral annular early velocity; E/A, early to late diastolic transmitral flow velocity; LVEF, left ventricular ejection fraction; ACEI, angiotensin-converting enzyme inhibitor; ARB, angiotensin II receptor blocker; CCB, calcium channel blockers*.

* *is compared with 0–2 group P < 0.05*,

ψ *is compared with 3–4 group P < 0.05*,

† *is compared with 5–6 group P < 0.05*.

**Figure 2:**
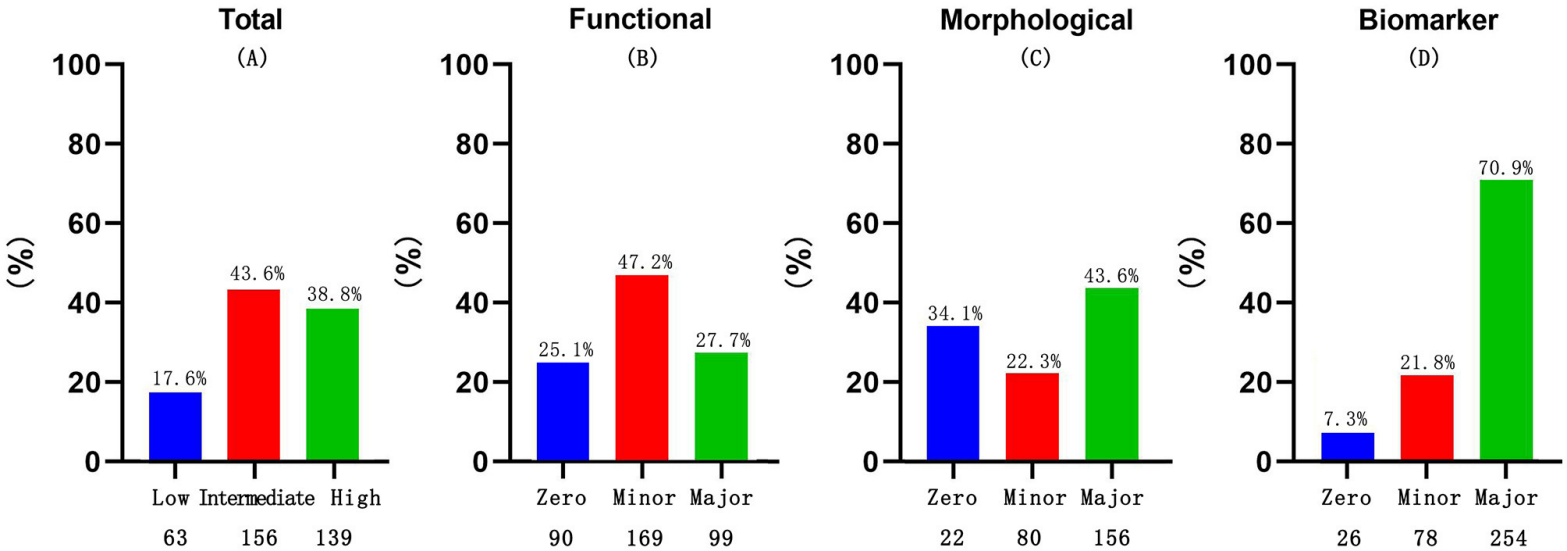
Discriminates between the major and minor criteria for total HFA-PEFF score categories **(A)**, functional **(B)**, morphological **(C)** and biomarker sub-scores **(D)**, among the cohort.

In the functional domain which included echocardiographic findings, nearly 50% of the patients received 1 point, whereas the majority of patients in the cohort met the major criterion in both the morphological and biomarker domains (Figure 2).

### Endpoint on Follow-Up

**Figure 3:**
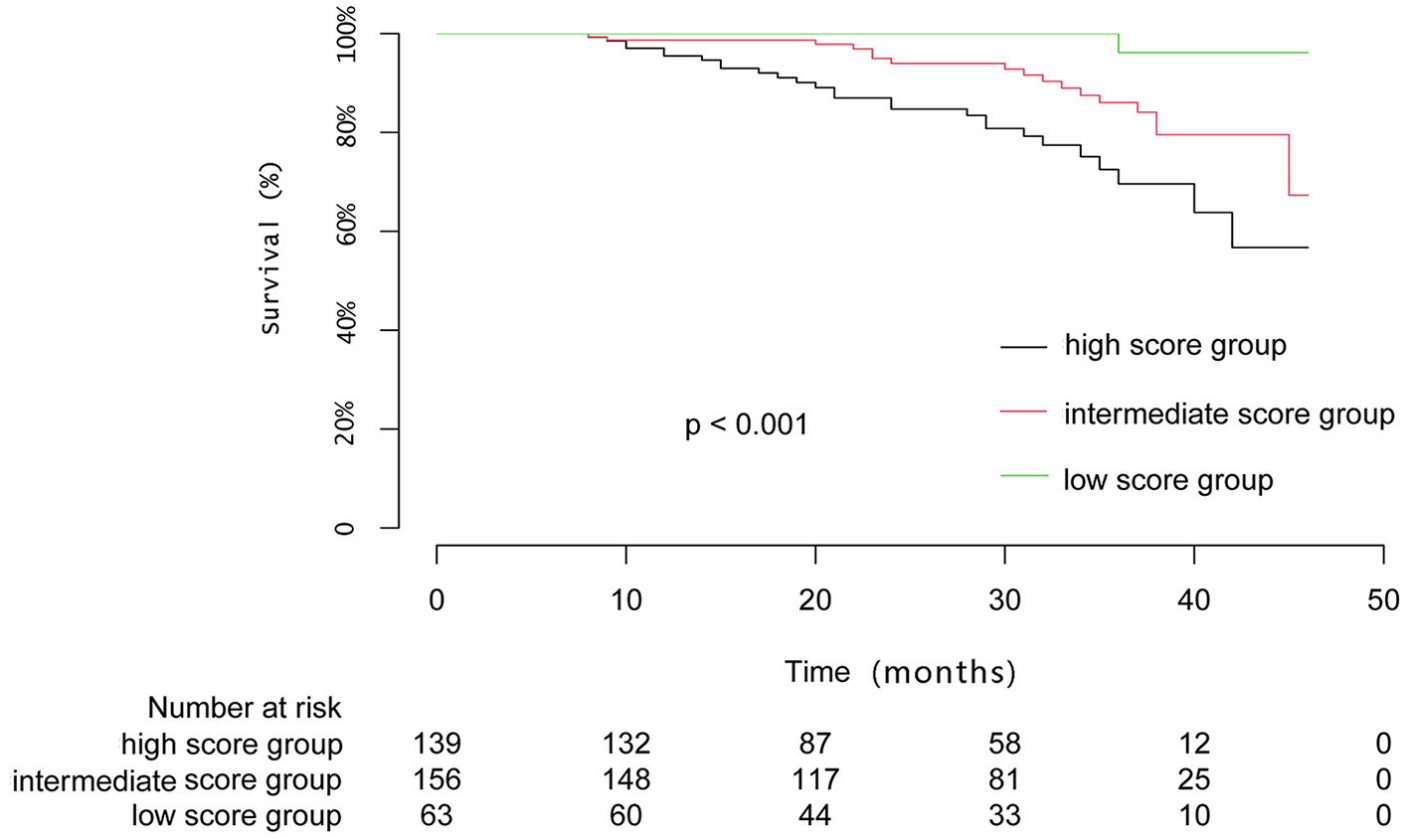
Mortality stratified by the low, intermediate, and high HFA-PEFF scores.

The patients were followed up for an average of 26.9 ± 11.1 months. Of the cohort, 46 patients died (12.8%), with rates for the low, intermediate, and high score groups of 1 (1.6%), 18 (11.5%), and 27 (19.4%), respectively. A Kaplan–Meier analysis was performed and the log-rank test showed the mortality rate of the high score group was significantly higher than the low (1.6%) and intermediate score groups (11.5%) (*P* < 0.001) (Figure 3). During the follow-up period, the mortality rate of the three groups significantly differed at 12 and 24 months following the discharge (*P* = 0.025) (Supplementary Table 1).

The multivariate Cox regression demonstrated that age (*HR*: 1.086, 95% *CI*: 1.029–1.146, *P* = 0.003), diabetes (*HR*: 2.915, 95% *CI*: 1.428–5.948, *P* = 0.003), UA (*HR*: 1.004, 95% *CI*: 1.002–1.006, *P* = 0.000), d-dimer (*HR*: 1.000, 95% *CI*: 1.000–1.000, *P* = 0.003), and HFA-PEFF score (*HR*: 1.314, 95% *CI*: 1.013–1.705, *P* = 0.039) were significant predictors of the all-cause mortality after being fully adjusted (Table 3). The intermediate score group (*HR*: 4.912, 95% *CI* 1.154–20.907, *P* = 0.031) and high score group (*HR*: 5.291, 95% *CI*: 1.239–22.593, *P* = 0.024) showed a higher risk of all-cause death compared with the low score group after adjustment (Table 4).

**Table 3:** Risk factors of all-cause death in heart failure with preserved ejection fraction (HFpEF).

	**Univariate analysis**			**Multivariate analysis**		
	**HR**	**95% CI**	***P***	**HR**	**95% CI**	***P***
HFA-PEFF score	1.449	1.151–1.823	0.002	1.314	1.013–1.705	0.039
Age	1.083	1.032–1.137	0.001	1.086	1.029–1.146	0.003
**NYHA class**						
III/II	2.884	0.871–9.554	0.083	1.574	0.463–5.354	0.467
IV/II	6.755	1.985–22.982	0.002	2.538	0.694–9.285	0.159
Diabetes	2.774	1.477–5.209	0.002	2.915	1.428–5.948	0.003
Hypertension	2.072	0.819–5.246	0.124	1.002	0.339–2.962	0.997
Atrial Fibrillation	1.830	1.003–3.340	0.049	1.343	0.658–2.737	0.417
Angina	1.542	0.594–4.005	0.374	1.044	0.354–3.086	0.937
Cre	1.003	1.001–1.005	0.003	1.002	0.999–1.005	0.274
UA	1.004	1.002–1.006	0.000	1.004	1.002–1.006	0.000
his-TNI	0.994	0.956–1.003	0.747	0.958	0.756–1.214	0.724
D-Dimer	1.000	1.000–1.000	0.000	1.000	1.000–1.000	0.003
LVEF	0.982	0.913–1.055	0.617	0.958	0.871–1.055	0.385

*NYHA, New York Heart Association; Cre, creatinine; UA, uric acid; hs-TNI, high sensitivity troponin; LVEF, left ventricular ejection fraction*.

**Table 4:** Risk of all-cause death in HFpEF.

	**Univariate analysis**			**Multivariate analysis**		
	**HR**	**95% CI**	***P***	**HR**	**95% CI**	***P***
Low score group	1.000	Reference	NA	1.000	Reference	NA
Intermediate score group	5.314	1.249–22.616	0.024	4.912	1.154–20.907	0.031
High score group	6.196	1.456–26.374	0.014	5.291	1.239–22.593	0.024

*NA, not applicable. Adjusted for age, NYHA Class, diabetes, hypertension, atrial fibrillation, angina, creatinine, uric acid, his-TNI, d-dimer, LVEF*.

**Figure 4:**
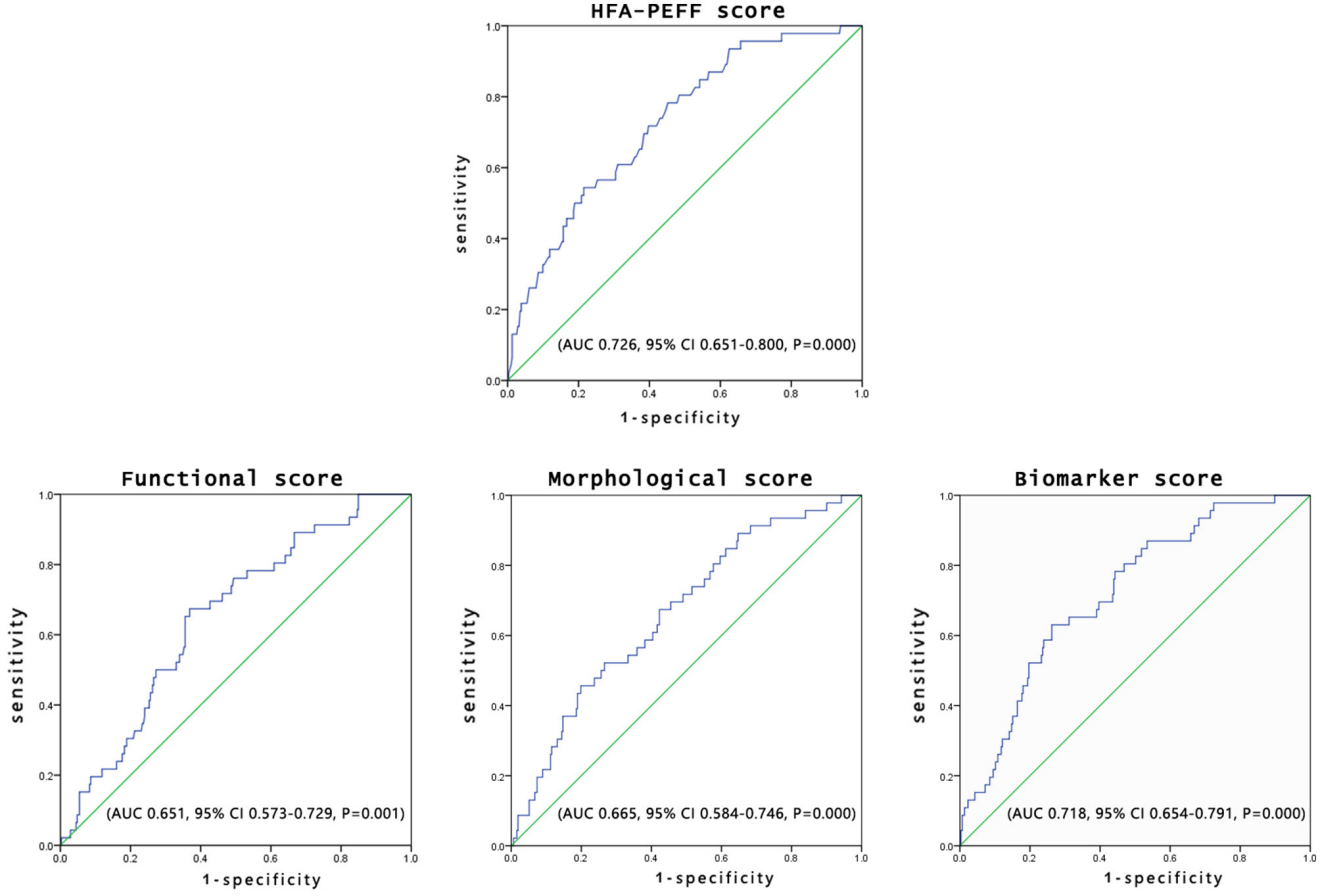
Prognostic performance of HFA-PEFF score and domain sub-scores.

Receiver operating characteristics analysis was constructed to evaluate the availability of HFA-PEFF score to predict the all-cause mortality in the patients with HFpEF, with an area under the curve (AUC) of 0.726 (95% *CI*: 0.651–0.080, *P* = 0.000) after adjusting for age and New York Heart Association (NYHA) class. The sensitivity and specificity of the HFA-PEFF score at the cut-off of 3.5 were 78.3 and 54.8%, respectively. Additionally, the functional sub-score showed relatively little prognostic value, whereas the biomarker sub-score reached an area under the curve similar to that of the total score, which was considered as the most significant parameter among the three sub-scores (Figure 4).

## Discussion

The main findings of this study are that: (1) HFA-PEFF score is not only a valuable diagnostic tool for HFpEF but can also effectively predict the prognosis. (2) A Cox regression analysis identified the HFA-PEFF score as an independent predictor of the all-cause mortality in the patients with HFpEF, and higher scores are associated with a higher incidence of adverse events. (3) The optimum cut-off value of the HFA-PEFF score is 3.5 for all-cause mortality.

### Outcomes in HFpEF

To date, the mortality of patients with HFpEF has been investigated in the clinical trials (13–16), observational studies (17, 18), and meta-analyses (19). However, the estimates of mortality varied considerably mainly due to the difference in the study design, baseline risk of the study population, and EF cut-off value used to define HFpEF (20). In-hospital, the mortality ranges from 2.4 (21) to 4.9% (22), with slightly higher 30-day (5%) (17) and 60–90 day (9.5%) (23) mortality. By 1-year post-diagnosis, the mortality varied from 20 to 29% (17, 19, 22, 24). By 5 years, approximately half of all the patients with HFpEF have died, with an estimated mortality ranging from 53 to 74% (19, 24–26). Currently, the cardiovascular causes remain a significant contributor to the mortality in patients with HFpEF (27). In this research, only 12.8% of patients with HFpEF died over ~2 years of follow-up, which is lower than the percentage reported elsewhere. This discrepancy may be attributed to the different baseline characteristics of prior cohorts, namely, older age, higher comorbidity burden, and worse cardiac function with frailer patients. These characteristics would predispose to the patients to higher rates of death (28–33).

### Comorbidities and Prognostic Factors in HFpEF

Heart failure with preserved ejection fraction is often accompanied by multiple cardiac and non-cardiac comorbidities, making the diagnosis and treatment more difficult. These comorbidities are often associated with a higher incidence of various adverse outcomes in HFpEF. In this cohort, the enrolled subjects also suffered a high burden of various comorbidities: hypertension (80.7%), diabetes (48.9%), AF (53.7%), and MI (23.5%). Surprisingly, these common comorbidities showed no positive correlation with all-cause death after being fully adjusted, except for diabetes. AF and HFpEF commonly coexist and share common epidemiology, pathophysiology, pathogenesis, and risk factors (34). Both the prevalence and incidence of AF are associated with the increased mortality in HFpEF (35). In this research, AF was not positively correlated with the all-cause mortality after adjustment, which is probably due to the relatively small sample and short follow-up period. Diabetes mellitus can increase the risk of morbidity and mortality in patients with chronic HF, and the negative prognostic association may be greater in HFpEF than in HFrEF. Diabetes mellitus in HFpEF may be accompanied by poorer functional status and lower exercise capacity, increased markers of inflammation/fibrosis/endothelial dysfunction, worse congestion, higher LV filling pressures, and increased mortality and HF admissions. The potential pathophysiological mechanisms include sodium retention, volume overload, metabolic disorders, systemic inflammation, poor skeletal muscle function, impaired peripheral oxygen delivery, and chronotropic incompetence (36).

A number of factors could affect the prognosis of HFpEF (37–41). LV diastolic dysfunction plays a central role in the pathophysiology of HFpEF, defined as an impairment in relaxation or an increase in stiffness. LV diastolic dysfunction can cause an elevation in LV filling pressure and promote the symptoms of dyspnea, can impair exercise capacity, and increase the risk of hospitalization and death (42, 43). In addition to LV diastolic dysfunction, multiple non-diastolic abnormalities may influence the prognosis of HFpEF, such as pulmonary hypertension (PH), right ventricular (RV) dysfunction, elevated plasma natriuretic peptide levels, and increased red cell distribution width (RDW). PH is common in HFpEF, seen in nearly 80% of the patients, and mortality is increased in this cohort (44). Pulmonary congestion is an important factor causing PH. Coiro et al. reported that the development of pulmonary congestion during exercise, as easily assessed by lung ultrasonography, is an independent predictor of cardiovascular death and heart failure rehospitalization in patients with HFpEF. In their research, the patients with B-line change > 10 or peak B-lines > 10, a semiquantitative indicator evaluating increased extravascular lung water, experienced a higher risk of adverse outcomes during the follow-up (45). Continued stimulation of PH can inescapably lead to RV systolic dysfunction, which is common and associated with adverse outcomes in patients with HFpEF (44). Recent research demonstrated the response of the coupling of RV to PH is significantly important and can be assessed by the ratio of tricuspid annular plane systolic excursion (TAPSE) and right ventricular systolic pressure (RVSP), a lower ratio always implies adverse events in HFpEF (46). The natriuretic peptides biomarkers (BNP and NT-proBNP) are hormones released in response to the increased cardiomyocyte stretch. The normal levels have been traditionally used to exclude the diagnosis of HF for patients presenting with dyspnea. Increased levels, by contrast, have been associated with a worse prognosis in HF. For example, elevated levels of BNP are associated with higher risks of mortality and HF rehospitalization both in the inpatients and outpatients over a follow-up of 12 months (47). RDW represents the variability of sizes of circulating erythrocytes and can be measured in a complete blood count. Recently, RDW has received more attention and has been known as an available biomarker to evaluate various cardiovascular disorders (48–51). A higher level of RDW has been associated with increased all-cause mortality in patients with acute HF with preserved but not with reduced LVEF (52). In addition, Imai et al. reported RDW levels at admission can independently predict the poor outcomes caused by non-cardiac events in the patients with acute decompensated HFpEF (53).

### Future Prospects for HFpEF

Before the publication of the PARALLAX trial, there were no definitive treatments proven to improve the prognosis of patients with HFpEF (54, 55). In 2020, ESC recommended the use of sacubitril/valsartan for HF across the full ejection fraction spectrum. The investigators found that sacubitril/valsartan at the target dose reduced the incidence of the first hospitalization due to HF and composite of time to death due to cardiac failure or HF hospitalization, as well as delaying estimated glomerular filtration rate (eGFR) decline. Additionally, regular sacubitril/valsartan use significantly reduced the level of NT-proBNP from the fourth week after taking the drug, indicating improvement of cardiac function. While these findings are encouraging, the subjects involved in the study were those with ejection fraction > 40% rather than ≥50%. Aside from the pharmacological treatment, there is evidence that aerobic exercise can improve the peak oxygen consumption and quality of life in patients with HFpEF (56). Recently, Ge et al. introduced a clinical phenotypic classification of HFpEF (57), which provides a better understanding of the risk factors, etiology, pathophysiology, and clinical course of HFpEF and contributes to guiding the targeted treatment. In the near future, targeting treatment to etiology and comorbidities may be another good choice in the treatment of patients with HFpEF (58). Machine learning (ML) algorithms have been used to identify the latent features otherwise not amenable to detection by the conventional techniques, and they have demonstrated promising utility for the diagnosis, management, and prediction of endpoints in HF. It is anticipated that they are more easily translated for clinical use in the near future (59).

### Limitations

Nevertheless, this study has several limitations. First, this was a retrospective, single-center study with a small number of subjects. Second, some functional parameters, such as mitral annular early diastolic velocity (e'), tricuspid regurgitation velocity, and LV global longitudinal systolic strain, were not routinely performed in our center and were only available in a small number of patients. Thus, these functional variables could not be explored as the potential prognostic factors, which may influence the predictive accuracy of functional sub-score.

## Conclusions

The HFA-PEFF score can be used to assess prognosis in the patients with HFpEF, and higher scores are associated with higher all-cause mortality.

## Data Availability Statement

The raw data supporting the conclusions of this article will be made available by the authors, without undue reservation.

## Ethics Statement

The studies involving human participants were reviewed and approved by the Institutional Review Board of Dalian Medical University. The Ethics Committee waived the requirement of written informed consent for participation.

## Author Contributions

YS was responsible for collecting clinical data and writing the paper. JS and JL assisted YS in collecting data and conducting telephone follow-up. MD, YZ, and XZ were responsible for the statistical analysis. EK helped address reviewers' comments. YX, YL, and GT were responsible for revising the paper and determining the research direction. All authors were involved in the drafting or revision of the manuscript.

## Funding

This work was supported in part by the National Science Foundation of China (No. 82170385, Recipient: YL).

## Conflict of Interest

The authors declare that the research was conducted in the absence of any commercial or financial relationships that could be construed as a potential conflict of interest.

## Publisher's Note

All claims expressed in this article are solely those of the authors and do not necessarily represent those of their affiliated organizations, or those of the publisher, the editors and the reviewers. Any product that may be evaluated in this article, or claim that may be made by its manufacturer, is not guaranteed or endorsed by the publisher.
