# Supplementary material for: Predictive Value of HFA-PEFF Score in Patients With Heart Failure With Preserved Ejection Fraction
Source: Front Cardiovasc Med. 2021 Oct 29;8:656536. doi: 10.3389/fcvm.2021.656536 (PMC8585787; doi:10.3389/fcvm.2021.656536)
Supplement: Supplementary file 1 [file Table_1.DOCX]

**Supplementary table 1.** Mortality at 12, 24, 36 and more than 36 months after discharge

| Mortality | 0-2points | 3-4points | 5-6 points | P |
| --- | --- | --- | --- | --- |
|  |  |  |  |  |
| Total | 1（1.6） | 18（11.5） | 27（19.4） | 0.002 |
| 0-12months（n,%） | 0（0） | 2（1.3） | 6（4.3） | 0.089 |
| 12-24months（n,%） | 0（0） | 5（3.2） | 11（7.9） | 0.025 |
| 24-36 months（n,%） | 1（1.6） | 6（3.8） | 8（5.8） | 0.376 |
| ＞36months（n,%） | 0（0） | 5（3.2） | 2（1.4） | 0.257 |
